# Cured by Extracting a Tooth

**Published:** 1855-06

**Authors:** C. A. Hathwell

**Affiliations:** Virginia, Cass Co., Ill.


ARTICLE III — *Cured by Extracting a Tooth.* By C. A. HATHWELL, M. D., of Virginia, Cass Co., Ill.

In a number of the N. W. Medical and Surgical Journal published some time back, I remember reading an article entitled "Cured by the Kick of a Horse." This instance of Horse Surgery prompted me to report the following case under the heading I have given it. It may not be wholly new, but I think it is somewhat of a novel case, and perhaps not altogether undeserving of a passing notice.

Sept. 14, 1854.—Was called to visit Miss S. D., residing in Clary's Grove, Menard Co. To use a legal phrase, she was a *spinster*, about 38 years of age. I found the following symptoms: High fever, redness of the face, throbbing of the temporals, pains in the back and extremities, cephalalgia, great anxiety and restlessness, skin dry, excessive thirst, tongue furred, breath hot and offensive, pulse full and voluminous, anorexia, bowels costive, urine scant but high colored and turbid, lips dry and parched. I also observed the left side of her face considerably swollen. In reply to the customary interrogatories of a physician to his patient, I received the ensuing history of the case, mainly from the lady's brother, Mr. D., in whose house she resided. He said she was plagued with the toothache very much,—that this was the third time she had been *attackted* in a similar manner. In a day or two after extreme suffering she would have a severe chill, then fever and the train of symptoms I have above related and the toothache would then subside. Upon receiving this information I proceeded to examine the lady's mouth (not though as we do a horse to see how old she was), but with a different intent. She opened her mouth with much difficulty, as the elevators were so contracted as to almost prohibit speaking to me. I discovered however the second inferior molar of the left side on its anterior surface perfectly decayed and gone below the alveolar, I told her she had better let me endeavor to extract the diseased tooth for her, as probably that was the source of her swelled face. Her reply was, that the former family physician, Dr. —— had considerable difficulty in drawing a tooth several months previous, and had informed her "that all her teeth would pull remarkably hard." Now I did not possess audacity sufficient to differ in opinion with a practitioner of such vast sagacity and profound judgment, so I urged her no further, but proceeded to prescribe.

Every physician is aware that several diseases are ushered in by similar symptoms to those I have given; but taking into consideration the periodical nature of the case, the mode of attack, coming on every other day with chills, etc., I considered it a case of Intermittent Fever, and gave—

℞ Hyd. Prot. Chlo., ℈jss.

Pulv. Doveri, gr. xv.

M. ft. in chart No. iii., one to be given every three hours, and afterwards the bowels to be freely evacuated with senna tea, hops stewed in vinegar applied warm to her swelled face, and diluent drinks.

Sept. 15.—Called again, her bowels had been sufficiently moved, no fever, swelling of the face diminished, etc. Seeing her in a condition for the exhibition of quinine, I prescribed that article, to be administered at appropriate intervals. I again asked the lady if I should then relieve her of her troublesome companion (the tooth), but permission was not granted, for "as long as it didn't ache, it could do no good to pull it." I then took my departure with the passing observation that I did not think there would be any necessity of my calling on her the next day.

Sept. 28.—Two weeks from the above date, a messenger called at my office and requested me to visit Miss D., "as she was down again. I obeyed, and on approaching the bedside and examining the case, I found her in the same situation, or nearly so, as when I had first seen her,—fever, face swollen, etc. She remarked she was glad to see me, and wished I would cure her up again. I was now thoroughly persuaded her decayed tooth was the primary cause of disease, and that her fever, etc., was exclusively symptomatic, so requested her to allow me to extract it. She consented, with the proviso that I would place her under the influence of chloroform. To this I made an objection, principally however because I did not happen to have any along with me; so she would not listen to having it pulled. Finding the previous treatment entirely successful, I repeated it, gave her two visits and discharg-. the case.

October 17.—Two weeks and four days from the last date, in the night, whilst I was comfortably enjoying myself in the arms of Morpheus, I was suddenly roused by a visit from the lady's brother, Mr. D., who thus classically expressed himself: "Doctor, I want you to come to our house right away, and jerk that cussed tooth out of Sarah's head. It has been aching again for two or three days, but it has stopped now and she is down sick again, jist like you seed her before: never mind what she says, but come at once and out with it." His tone and countenance convinced me that he was resolute, and it would be in vain for me to solicit him to wait till morning, so off we rode upon our way rejoicing.

As I expected, my patient was in bed, and as I had before noticed her, nervous and irritable, respiration difficult and hurried, fever high, pulse rapid, etc. She stated that, as soon as her tooth ceased aching, her general health suffered, and she was resolved to have it out at last, as she was perfectly satisfied it was the sole origin of all her trouble and distress. I promptly removed it, and cannot truthfully say, with my keen professional brother, that it "pulled so remarkably hard" as he had so sagaciously predicted. It is unnecessary to record her expressions of thankfulness after I had *jerked it out*. The roots of the tooth were materially absorbed, suppuration had commenced, as a little pus was discharged from the cavity. As her bowels were constipated, I left her some purgative pills, a few seidlitz powders, and directed her to take quinine during Apyrexia, and remarked I was now in hopes she would have no occasion to send for me again, and then very willingly made my exit.

In February last I was summoned to attend a patient in the same neighborhood, and through motives of curiosity I made a casual call upon my inveterate tooth case. I found her (as she said) enjoying better health than for some years antecedent to the pulling of the tooth, had never any unpleasant symptoms since my departure, and only wished her and her former enemy had parted company long before they did.

Such is the history of a rather uncommon case. I have been minute in its details, and, the reader may imagine, too prolix; but I could not condense it satisfactorily and present it as it actually occurred. Taking into consideration its periodical nature, its deceptive tendency, and concomitant circumstances. I deem it worthy of record, and may perhaps, like a beacon to the mariner, prove a guide to another practitioner, and enable him to afford relief to some unfortunate sufierer. I am not aware that I can christen it with a more approprate *soubriquet* than "Cured by extracting a tooth."

